# Toripalimab plus chemotherapy in the treatment of metastatic triple-negative breast cancer: a cost-effectiveness analysis

**DOI:** 10.3389/fpubh.2024.1421826

**Published:** 2024-07-29

**Authors:** Hongfu Cai, Lisheng Huang, Zhiwei Zheng

**Affiliations:** ^1^Department of Pharmacy, Fujian Medical University Union Hospital, Fuzhou, China; ^2^Department of Radiation Oncology, Cancer Hospital of Shantou University Medical College, Shantou, China; ^3^Department of Pharmacy, Cancer Hospital of Shantou University Medical College, Shantou, China

**Keywords:** metastatic triple-negative breast cancer, toripalimab, chemotherapy, cost-effectiveness analysis, partitioned survival model

## Abstract

**Objective:**

This study focuses on assessing the cost-effectiveness of incorporating toripalimab alongside chemotherapy for the treatment of patients diagnosed with metastatic triple-negative breast cancer from the perspective of the Chinese healthcare system.

**Methods:**

A partitioned survival model was constructed to simulate the costs and health outcomes over the lifetime of patients with mTNBC. Clinical data regarding overall survival, progression-free survival, and treatment-related adverse events were derived from the TORCHLIGHT clinical trials. Incremental cost-effectiveness ratio (ICER) were calculated based on the gains in quality-adjusted life-year (QALY). The willingness-to-pay (WTP) threshold was defined as $39,855.79 per QALY. Additionally, sensitivity analyses were conducted to examine the robustness of the model.

**Results:**

The total cost incurred by the group receiving toripalimab was $38,040.62, while the placebo plus chemotherapy was $26,102.07. The utilization of the toripalimab regimen resulted in an increase of 0.74 QALYs and an incremental cost of $11,938.55 compared to the placebo plus chemotherapy group. The ICER was $16,133.18/QALY, indicating that toripalimab plus chemotherapy is a cost-effective strategy according to the WTP threshold. Sensitivity analyses confirmed the robustness of the results.

**Conclusion:**

This study suggests that the addition of toripalimab to chemotherapy for the treatment of mTNBC is a cost-effective strategy. The findings provide valuable evidence to guide decision-making regarding treatment selection for patients with mTNBC in China.

## 1 Introduction

Breast cancer stands as the most prevalent form of cancer among women, with a global tally of 2,308,897 new cases in 2022, constituting 11.6% of all newly diagnosed tumors and ranking second amongst all cancer types (1). Triple-negative breast cancer (TNBC) is a highly concerning subtype of breast cancer that is characterized by the absence of key receptors, including the estrogen receptor (ER), progesterone receptor (PR), and human epidermal growth factor receptor 2 (HER2) (2). This specific feature poses a significant clinical challenge as it limits the availability of specific therapeutic targets, beyond the conventional chemotherapy treatment approach. Despite accounting for only 15–20% of all breast cancers, TNBC has been associated with the poorest prognosis compared to other breast cancer subtypes (3). Systemic chemotherapy regimens that include taxanes and platinums have traditionally been considered the gold standard first-line treatment for TNBC before the advent of immunotherapy (4). However, despite the diligent efforts in treatment strategies, the median overall survival for individuals diagnosed with metastatic TNBC (mTNBC) remains dishearteningly low. Moreover, it is crucial to emphasize that the present 5-year survival rate for TNBC stands at a mere 12% (5), underscoring the pressing requirement for innovative therapeutic interventions capable of adequately tackling this substantial unmet medical demand.

In recent years, the advent of immunotherapy has brought about renewed hopes for enhancing treatment outcomes in TNBC (6, 7). One of the groundbreaking immunotherapy strategies in TNBC involves the use of immune checkpoint inhibitors (ICIs). These inhibitors target key molecules, such as programmed cell death protein 1 (PD-1) and programmed death-ligand 1 (PD-L1), that regulate immune responses and prevent excessive immune activation. By blocking these checkpoints, immune checkpoint inhibitors restore and enhance anti-tumor immune responses, leading to improved control and eradication of cancer cells. In the IMpassion130 trial, the combination therapy of atezolizumab and nab-paclitaxel was found to significantly extend the duration of time progression-free survival (PFS) in both the subgroup of patients with positive expression of the PD-L1 protein and in the overall study population (8). Similarly, the KEYNOTE-355 study demonstrated that pembrolizumab, when used in combination with chemotherapy, resulted in significant improvements in both PFS and overall survival (OS) compared to chemotherapy alone. These improvements were specifically observed in TNBC patients with a combined positive score (CPS) of PD-L1 expression ≥10 (9). These important clinical trials provide strong evidence supporting the use of immunotherapy in combination with chemotherapy as an effective therapeutic approach for TNBC patients.

Recently, Jiang et al. conducted a significant multicenter, randomized, double-blind phase 3 trial, referred to as TORCHLIGHT, which evaluated the efficacy of combining toripalimab and nab-paclitaxel compared to placebo plus chemotherapy in patients with mTNBC (10). The use of toripalimab, a novel immune checkpoint inhibitor, in combination with nab-paclitaxel provided a rationale for investigating the potential synergistic effects of this therapy. The median PFS was observed to be 8.4 months in the experimental arm, while it was 5.6 months in the control arm. Furthermore, this combination therapy also showed a notable improvement in median overall survival, with a median OS of 32.8 months in the experimental arm compared to 19.5 months in the control arm. This study demonstrated a significant improvement in median progression-free survival (mPFS) by 2.8 months in the experimental group, along with a notable 35% decrease in the risk of disease progression or mortality. Additionally, a descriptive analysis of OS indicated a favorable trend in both the PD-L1-positive and intention-to-treat (ITT) populations. Importantly, the incidence of treatment-emergent adverse events (AEs) in the experimental arm was comparable to that in the control arm, indicating that the addition of toripalimab to nab-paclitaxel did not significantly increase the risk of side effects. These findings suggest that the inclusion of toripalimab in the treatment regimen holds promise for PD-L1-positive patients with mTNBC, as it leads to a significant enhancement in PFS and potentially prolongs overall survival.

However, despite the encouraging initial findings, there is a notable dearth of comprehensive evaluations concerning the cost-effectiveness of the combination therapy involving toripalimab and nab-paclitaxel in comparison to chemotherapy administered as a standalone treatment. Considering the limited treatment alternatives and the potential for enhanced outcomes associated with the use of toripalimab in mTNBC, it becomes crucial to gain a better understanding of the cost-effectiveness of this intervention. In this study, our objective is to scrutinize the incremental cost-effectiveness ratio (ICER) of toripalimab plus nab-paclitaxel when contrasted with chemotherapy administered as the sole treatment. This analysis will furnish vital information regarding the additional expenses incurred to achieve an additional unit of health benefits. The findings generated from this investigation will not only provide valuable insights for healthcare practitioners and policymakers but also facilitate resource allocation decisions and contribute to the optimal management of this disease.

## 2 Methods

### 2.1 Model establish

**Figure 1:**
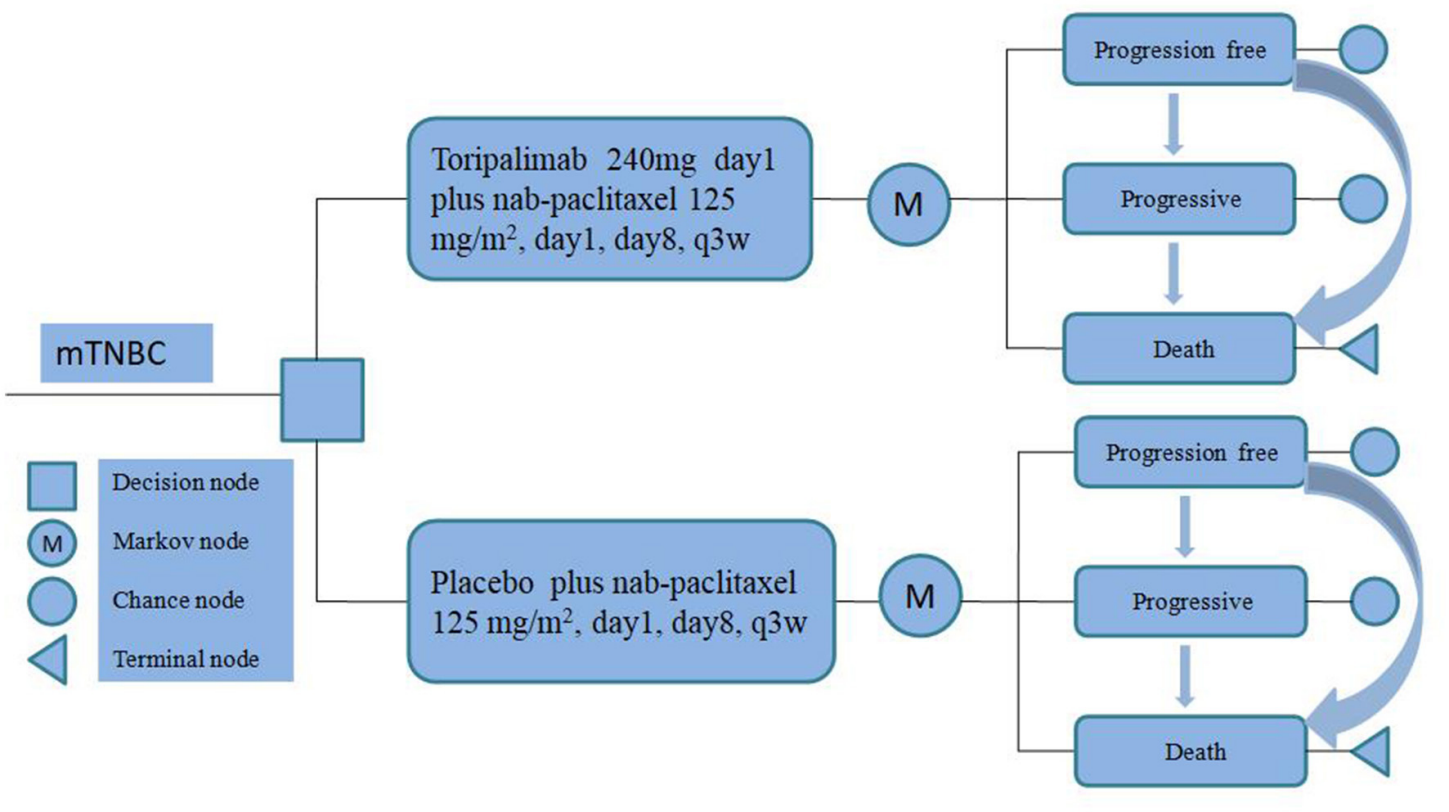
Partitioned survival model.

To evaluate the cost-effectiveness of toripalimab, we utilized a partitioned survival model (PSM) to accurately simulate the progression of the disease and treatment outcomes over a 10-year time horizon for mTNBC. Our PSM categorizes patients into three distinct and mutually exclusive states: progression-free disease (PFD), progressive disease (PD), and death (Figure 1).

In order to align with the duration observed in the TORCHLIGHT clinical trial, we implemented a simulation cycle period of 21 days. All expenses associated with this study were converted into US dollars (USD) by utilizing the average exchange rate from RMB (Ren min Bi) to USD in the preceding year (2023), which stood at 100 USD per 705 units of RMB (11).

In line with established economic evaluation frameworks, we considered a willingness-to-pay (WTP) threshold of $39,855.79 per QALY according to the latest <<The China Guidelines for Pharmaceutical Economics Evaluation 2020>> as a significant criterion in China. This threshold holds particular significance as it is set at three times the national gross domestic product (GDP) for the year 2023 (12). By incorporating the WTP threshold, our intervention's cost-effectiveness was assessed by evaluating the incremental cost per additional QALY gained. The construction of the PSM was accomplished through the utilization of the TreeAge Pro 2011 software application.

### 2.2 Model population and treatment

The present model posits the underlying assumption that the target population under investigation exhibits a congruous composition of participants as that observed within the TORCHLIGHT trial. A total of 353 female patients were randomly assigned to the experimental arm, while 178 patients were assigned to the control arm. All participants in this study were of Asian ethnicity and had a median age of 53 years (ranging from 23 to 84 years) and 54.5 years (ranging from 27 to 76 years) in the toripalimab and placebo plus chemotherapy groups, respectively. The allocation of patients based on their PD-L1 expression levels was evenly distributed between the two treatment groups. Specifically, 30.6 and 34.8% of patients in the toripalimab and placebo plus chemotherapy groups, respectively, exhibited PD-L1 CPS expression levels below 10. Moreover, 26.1 and 21.3% of patients in the toripalimab and placebo plus chemotherapy groups, respectively, demonstrated PD-L1 CPS expression levels of 10 or higher.

Participants will be subject to random allocation for administration of either toripalimab (240 mg, day 1) in conjunction with nab-paclitaxel (125 mg/m^2^, day 1, day 8) every 3 weeks or a placebo plus chemotherapy in combination with nab-paclitaxel until the point of disease progression or the occurrence of intolerable adverse effects.

In order to enhance the efficiency of the modeling process, the current study primarily focuses on examining and comparing grade 3 or 4 adverse events that manifest at a frequency surpassing 4% in the cohorts.

In the toripalimab group, the median duration of therapy for the treated population was 21.14 (0.14–129.42) weeks, while in the placebo plus chemotherapy group, it was 22.14 (0.14–125.57) weeks. A total of 74 patients (21.0%) in the toripalimab cohort and 38 patients (21.3%) in the placebo plus chemotherapy cohort received subsequent anticancer therapy. In order to conduct a cost-effectiveness analysis, we made the assumption of using a second-line treatment consisting of gemcitabine plus carboplatin chemotherapy regimen for both treatment groups. However, considering the significant uncertainty surrounding the optimal choice of third-line therapy, our study assumes the utilization of the best supportive treatment regimen in case of disease re-progression.

### 2.3 Clinical transfer probability data extraction

In this study, we have conducted a rigorous analysis of the data acquired from the TORCHLIGHT clinical trial, utilizing the widely recognized data extraction tool GetData Graph Digitizer software. The primary aim of this study was to meticulously extract and subsequently model the survival curves, encompassing both overall survival and disease-free survival measures, by employing a rigorous process of identifying the statistically optimal distributions for these variables.

To determine the optimal Data reconstruction and best-fit survival curves, we employed a combination of two fundamental principles. Firstly, we employed the Akaike Information Criterion (AIC) and the Bayesian Information Criterion (BIC) as the minimum statistical criteria (13). These statistical criteria enabled us to evaluate the goodness-of-fit of various distributions to the data and determine the most suitable models. The AIC and BIC values associated with the simulated survival curves can be found in Supplementary Table 1. Secondly, we conducted an intuitive visual inspection to ensure that the simulated curves aligned with the clinical trial. Supplementary Figure 1 presents a graphical representation of the reconstructed distribution curves for each respective group. Ultimately, the log-logistic distribution was conclusively found to provide the optimal fit for the purpose of simulating survival curves.

In order to improve the effectiveness of our model, a simulation approach was implemented to generate survival times based on the log-logistic distribution. This innovative technique allowed us to extend the potential applicability of our model beyond the duration of the clinical trial follow-up. By providing a robust estimation of the survival function, denoted as S(t), we aimed to offer a more comprehensive understanding of the underlying dynamics of the population under study. In our study, we employed the log-logistic distribution to model the survival function, which can be expressed as S(t) = 1/(1 + λt^γ^) (20). This parametric distribution plays a crucial role in survival analysis due to its flexibility in capturing various shapes of survival curves. The estimated values of the parameters, namely shape (γ) and scale (λ), are presented in Table 1.

**Table 1:** The parameters input of the model.

**Parameters**	**Input value**	**Range**		**Distribution**	**References**
		**Minimum**	**Maximum**		
**Log-logistic PFS survival model**					
Toripalimab group	γ = 1.53; λ = 0.033	-	-	-	(10)
Placebo plus chemotherapy group	γ = 2.01; λ = 0.024	-	-	-	(10)
**Log-logistic OS survival model**					
Toripalimab group	γ = 1.69; λ = 0.0027	-	-	-	(10)
Placebo plus chemotherapy group	γ = 1.88; λ = 0.0030	-	-	-	(10)
**TEAE rate of toripalimab group (%)**					
Leukopenia	25.20	-	-	Beta	(10)
Neutropenia	26.30			Beta	(10)
Anemia	4.00	-	-	Beta	(10)
**TEAE rate of placebo plus chemotherapy group (%)**					
Leukopenia	23.40	-	-	Beta	(10)
Neutropenia	28.60	-	-	Beta	(10)
Anemia	2.90	-	-	Beta	(10)
**Drug cost ($)**					
Toripalimab (240 mg)	267.36	200.52	334.20	Gamma	(14)
Nab-paclitaxel (100 mg)	105.96	79.47	132.45	Gamma	(14)
Subsequent therapy per cycle ($)	137.61	103.21	172.01	Gamma	(14)
**Cost of TEAE per cycle ($)**					
Leukopenia	104.95	78.71	131.19	Gamma	(15)
Neutropenia	547.50	410.63	684.38	Gamma	(16)
Anemia	607.06	455.30	758.83	Gamma	(16)
Best supportive care ($)	359.00	269.25	448.75	Gamma	(16)
Follow-up cost per cycle ($)	170.00	127.50	212.50	Gamma	(17)
**Utility**					
Leukopenia	0.09	0.07	0.11	Beta	(17)
Neutropenia	0.09	0.07	0.11	Beta	(17)
Anemia	0.12	0.09	0.15	Beta	(17)
Progression-free disease	0.76	0.57	0.95	Beta	(18)
Progressive disease	0.55	0.41	0.69	Beta	(18)
Discount rate	0.05	0.00	0.08	Beta	(19)
Body surface area (m^2^)	1.72	1.29	2.15	Beta	(16)

### 2.4 Cost and utility

Our model offers a comprehensive framework for evaluating the economic implications of treatment for mTNBC by encompassing a diverse range of significant direct healthcare expenditures. These expenditures include medication costs, management of adverse events, follow-up therapeutic interventions, and optimal supportive care.

In order to obtain precise information regarding drug costs, we obtained national median drug prices from the China Data Platform (https://data.yaozh.com/). These prices were then utilized as inputs for the analysis of drug prices in our study. Additional costs were derived from relevant literature sources that have been previously published. In this study, utility values ranging from 0 to 1 were employed to assess the quality of life associated with health status. However, we were unable to obtain explicit utility value data from the TORCHLIGHT clinical trial. Therefore, we resorted to obtaining utility values from previously published literature. It is important to note that these cost and utility values obtained from the literature are incorporated into our sensitivity analysis, which aims to assess the robustness of our model's results by examining their impact on the conclusions drawn from our findings. Furthermore, our model also considers the negative utility associated with adverse drug events. Table 1 provides comprehensive information on the cost and utility values.

### 2.5 Sensitivity analysis

This study employed sensitivity analysis to bolster the robustness of the model. A one-way sensitivity analysis was performed, wherein each input parameter was altered by ±25% to evaluate the influence of these parameters on the ICER. Furthermore, the discount rate was varied from 0 to 8%. The findings of this sensitivity analysis were graphically presented using tornado diagrams.

In order to rigorously evaluate the uncertainty surrounding the estimation of ICERs in this study, a Probabilistic Sensitivity Analysis (PSA) was also performed. A total of 1,000 Monte Carlo simulations were conducted in this analysis. The primary objective of the PSA was to randomly sample input parameters from specific probability distributions. This approach enables a comprehensive and robust assessment of the potential variability in the estimated ICERs, while considering the inherent uncertainty associated with each parameter. By drawing samples from designated probability distributions, the PSA not only considers deterministic parameter values but also incorporates probabilistic elements. The results of the probabilistic sensitivity analysis are visually presented through scatter plots.

## 3 Results

### 3.1 Base case cost-effectiveness results

The study's findings revealed that the total expenditure for the group receiving toripalimab was $38,040.62, while the placebo plus chemotherapy group incurred a total cost of $26,102.07. Additionally, the toripalimab regimen led to an increase of 0.74 QALYs compared to the placebo plus chemotherapy group. However, it should be noted that this additional benefit came with an incremental cost of $11,938.55. Consequently, the ICER was calculated to be $16,133.18 per QALY gained. Importantly, this ICER value is lower than the WTP threshold of $39,855.79 per QALY in China, suggesting that the use of the toripalimab regimen can be considered cost-effective within the Chinese healthcare system. Table 2 presents a summary of the findings gleaned from this analysis.

**Table 2:** The results of cost-effectiveness.

**Base case**	**Toripalimab plus chemotherapy group**	**Placebo plus chemotherapy group**
Cost ($)	38,040.62	26,102.07
QALYs	2.47	1.73
Incremental cost ($)	11,938.55	NA
Incremental QALY	0.74	NA
ICER ($/QALY)	16,133.18	NA

### 3.2 Sensitivity analysis on the cost-effectiveness results

**Figure 2:**
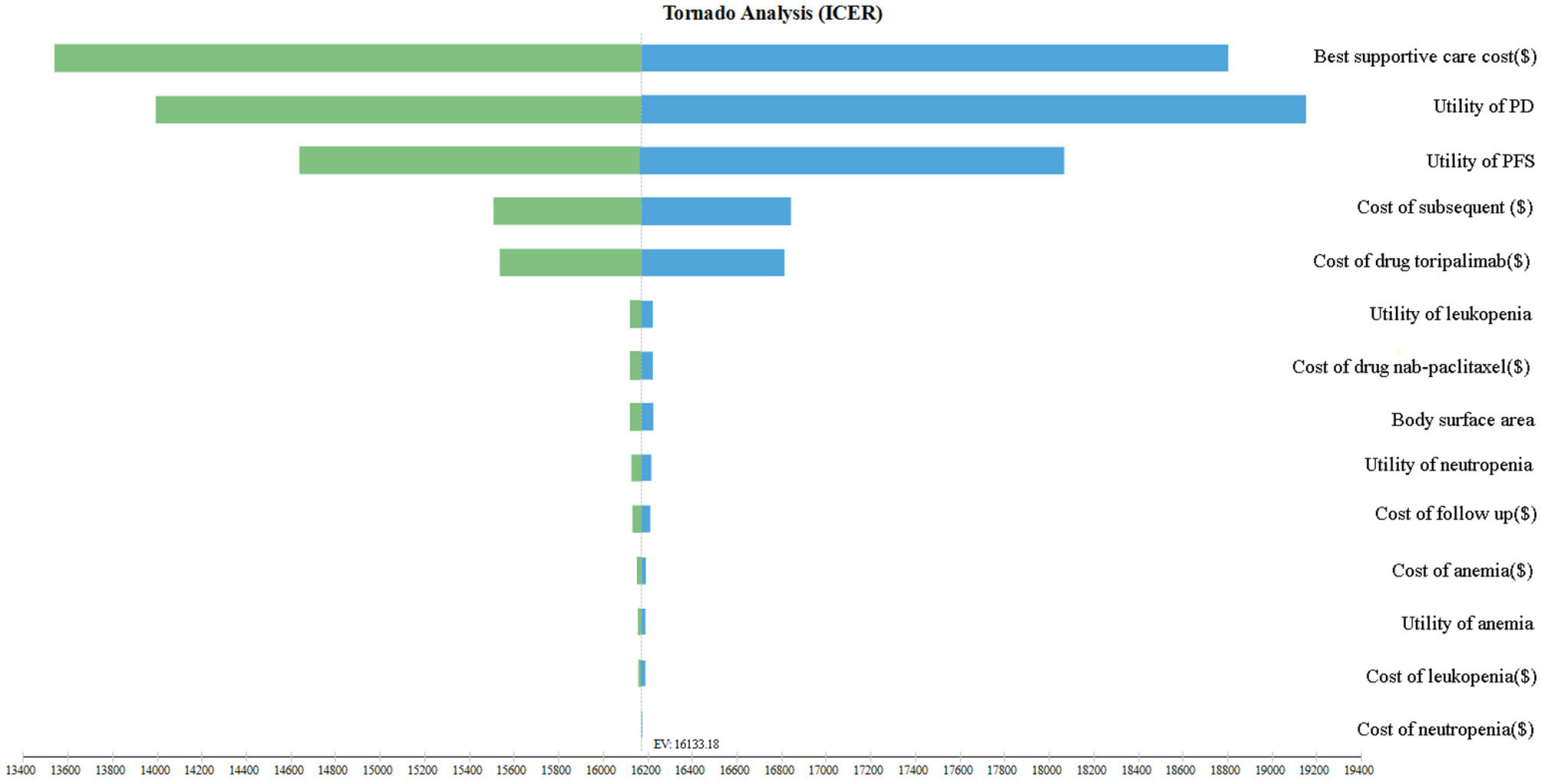
The tornado diagram.

Figure 2 presents a tornado diagram that showcases the results of a one-way sensitivity analysis. The most prominent factor affecting the ICER was found to be the cost of the best supportive care. However, it is worth noting that this impact fluctuated within a range of ±25%, which remains significantly lower than the designated threshold of WTP. Importantly, such fluctuations did not overturn the study's findings. Furthermore, other parameters such as PD utility, PFS utility, and subsequent therapy costs played a role in influencing the ICER; however, their impact gradually diminished. It is crucial to underscore that altering these parameters within a range of ±25% did not lead to substantial changes in the analysis results. Consequently, the persistent finding that the ICER value consistently remains below three times the GDP strengthens the stability and reliability of our findings.

**Figure 3:**
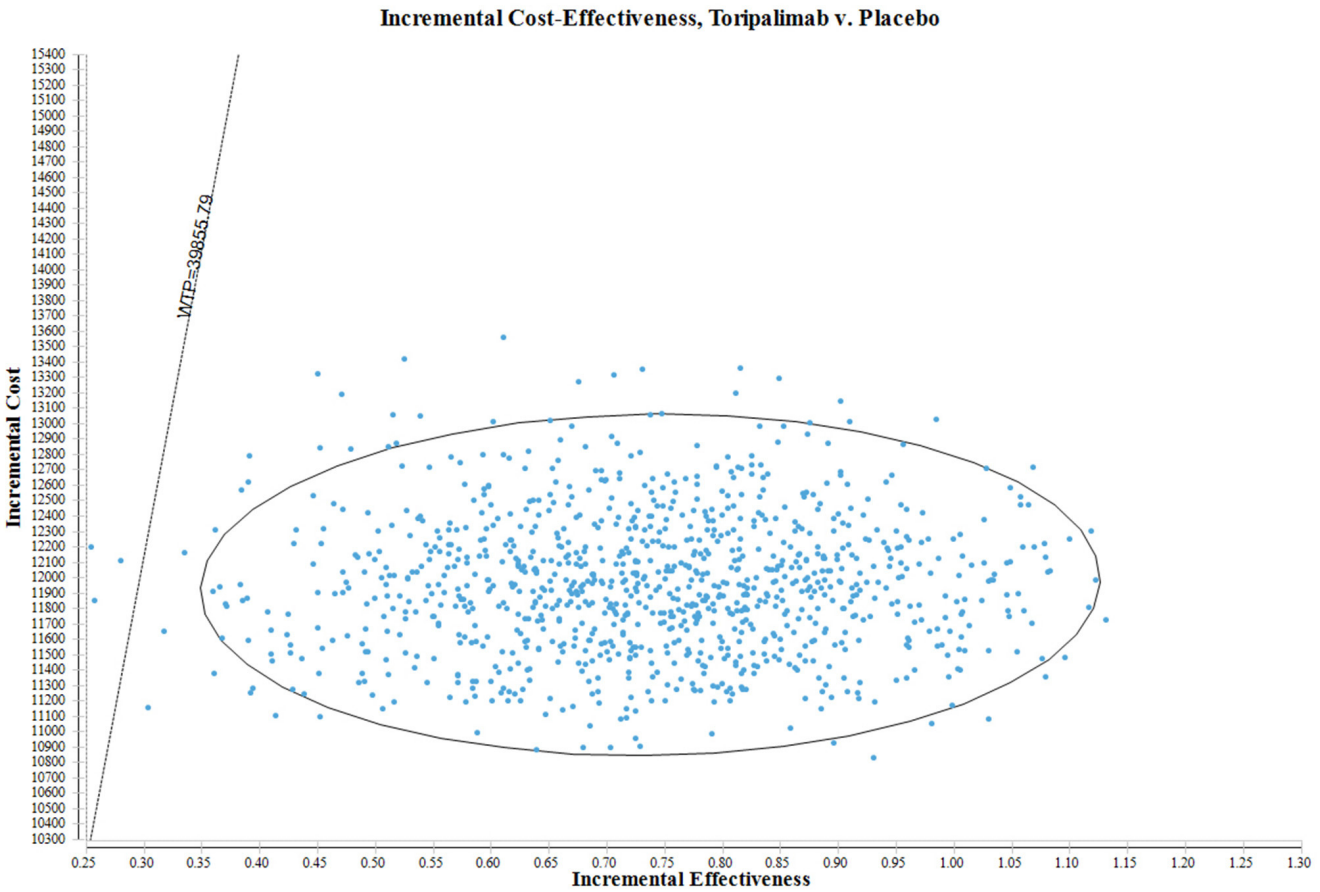
The scatter plot of PSA.

In Figure 3, the ICER plane provides a comprehensive illustration of the dispersion of the 1,000 bootstrap replicates of the ICER. This graphical representation provides valuable insights into the cost-effectiveness of different interventions. Based on the findings obtained from the analysis, interventions falling below the linear ICER line are considered to be cost-effective. This positioning implies that interventions in this quadrant exhibit a more favorable ICER ratio, indicating lower costs or greater effectiveness compared to interventions in other quadrants. Notably, considering the WTP threshold of $39,855.79 per QALY, there is a 99.60% probability of classifying the toripalimab regimen as a more cost-effective option in comparison to the placebo plus chemotherapy group.

## 4 Discussion

In recent years, China has achieved notable advancements in the field of cancer treatment, particularly in the development of innovative PD-1 or PD-L1 inhibitors (21). These inhibitors have demonstrated remarkable efficacy in enhancing the survival rates and clinical tolerance of individuals affected by various forms of cancer, significantly augmenting the prospects of cancer treatment (22). The centralized price negotiation mechanism has played a pivotal role in facilitating this positive trend, aiming to enhance the accessibility and affordability of these therapeutic interventions for patients (23). Furthermore, the implementation of a prioritized approval process in China has expedited the development, review, and approval of novel drugs. These initiatives have not only accelerated the introduction of innovative therapeutic agents but have also enabled timely access to treatment for patients in dire need (24). Consequently, these advancements in cancer treatment have not only positively impacted the lives of cancer patients in China but have also garnered global attention.

Toripalimab is a selective, recombinant, humanized monoclonal antibody against PD-1 (25). The TORCHLIGHT study aimed to compare the efficacy of toripalimab plus nab- paclitaxel vs. placebo plus chemotherapy as a first-line treatment for mTNBC. The study demonstrated that the mPFS was significantly improved by 2.8 months in the experimental arm, and there was a notable 35% reduction in the risk of disease progression or death. Moreover, a descriptive analysis of OS indicated a favorable trend in both the PD-L1-positive and ITT populations. These findings provide further validation for the clinical utility of incorporating PD-1 checkpoint blockade alongside chemotherapy to treat mTNBC. Our research conducted a rigorous and comprehensive cost-effectiveness analysis utilizing the ICER as a valuation metric to assess the affordability and value of toripalimab for the treatment of mTNBC.

To assess the cost-effectiveness of toripalimab, we employed a partitioned survival model to simulate the progression of disease and treatment outcomes over a 10 years time horizon. The model incorporated data from clinical trials and published literatures to estimate the outcomes and costs associated with toripalimab treatment. Our findings demonstrated that toripalimab was associated with a higher efficacy, indicated by improved the QALY, compared to the placebo plus chemotherapy treatment options. The combination therapy of toripalimab in conjunction with nab-paclitaxel has demonstrated a highly favorable ICER of $16,133.18 per QALY gained for the management of mTNBC. This ICER value is significantly lower than the WTP threshold of $39,855.79 per QALY. As a result, it suggests that incorporating the use of toripalimab plus nab-paclitaxel as a first-line treatment option for mTNBC in China has the potential to be deemed a cost-effective approach.

Our study also conducted sensitivity analyses to investigate the potential influence of changes in input variables on the outcomes of the study and model. By delving into the sensitivities of various parameters, we aimed to gain a deeper understanding of the interdependencies and the response of study results to individual factors. Notably, we identified several key factors that had a significant impact on the ICER, including the cost of the best supportive care, utility values for PFS and PD, and subsequent therapy costs. Importantly, we consistently observed that the resulting ICERs remained below the WTP thresholds, even when we varied all input parameters within a range of ±25%. This finding indicates that the use of toripalimab for mTNBC is a cost-effective strategy in China.

Currently, there are some research efforts are being dedicated to conducting economic evaluations of immunotherapies for patients with mTNBC. For example, a study by Liu et al. used a cost-effectiveness analysis to compare adding atezolizumab to paclitaxel for advanced or metastatic TNBC. The findings of this study indicated that adding atezolizumab to nab-paclitaxel is not a cost-effective strategy compared to nab-paclitaxel monotherapy for Chinese patients with advanced or metastatic TNBC in China (26). Another study by Lang et al. focused on evaluating sacituzumab govitecan compared with standard of care chemotherapy from the United States payer perspective. Their results demonstrated that sacituzumab govitecan at current price is unlikely to be a preferred option for patients with advanced or metastatic TNBC at a threshold of $ 150,000/QALY (27).

Cost-effectiveness analyses are essential tools for evaluating the value of therapies in healthcare (28). These analyses involve comparing the costs and outcomes of various treatment options, allowing healthcare professionals and policymakers to make informed decisions based on the available evidence. One crucial aspect that cost-effectiveness analyses assess is the impact of the therapy on health outcomes. Improved health outcomes are the ultimate objective of any treatment (29). Our study has concluded that the cost-effectiveness analyses demonstrate favorable ICER. This indicates that the utilization of toripalimab can substantially enhance health outcomes at a justifiable cost. This finding provides strong support for advocating the inclusion of toripalimab in treatment guidelines for mTNBC and for shaping healthcare reimbursement policies. By integrating toripalimab into treatment guidelines, healthcare providers can ensure that patients have access to a therapy that has proven to be both effective and cost-effective.

The correlation between the cost per QALY and a country's GDP per capita is a pivotal factor in determining cost-effectiveness thresholds. This association defines the threshold point at which the acquisition cost of a QALY becomes a determining factor in evaluating the value and feasibility of a certain intervention or policy decision within a given healthcare system (30). The concept of WTP per QALY encapsulates the notion that individuals are willing to assign a specific monetary value to acquire an additional year of life in perfect health or experience substantial enhancements in their quality of life (31). By quantifying this willingness to pay, policymakers and healthcare decision-makers gain a deeper insight into the relative value of various healthcare interventions and can apportion limited resources in a prudent manner. In this study, we have employed a threshold for the WTP criterion, which is set at 3 times the GDP, equivalent to $39,855.79 per QALY. This threshold aligns with the guidelines provided by the Chinese Pharmacoeconomics Guidelines. The ICER for toripalimab, when compared to placebo plus chemotherapy, was estimated to be $16,133.18 per QALY gained. Importantly, the ICER value we obtained is considerably lower than the generally accepted WTP threshold of $39,855.79 per QALY. This result suggests that the utilization of toripalimab as a first-line treatment for mTNBC has the potential to be considered economically efficient. Furthermore, even in the current context where some researchers and scholars propose reducing the WTP threshold to 1.5 times the GDP (32), our study demonstrates that the use of toripalimab for mTNBC would still be cost-effective in China.

It is crucial to acknowledge the limitations inherent in our study. Firstly, it should be noted that the data we have utilized for this cost-effectiveness analysis primarily originates from clinical trials. Nonetheless, it is imperative to acknowledge the necessity of ongoing monitoring and updating of these findings. As new evidence emerges and the landscape of cost and efficacy evolves, it becomes vital to continuously reassess and revise our conclusions. Secondly, it is crucial to acknowledge the assumption made in this study regarding the cost of the best supportive care and second-line treatments after disease progression. However, it is worth considering that in real word situations, the selection of best supportive care and subsequent treatment options would vary depending on the unique circumstances of each patient. Fortunately, the one-way sensitivity analyses conducted in this study provide reassurance as they consistently demonstrate that even when the estimated ranges for the cost of best supportive care and subsequent treatments are adjusted, the ICER values remain below the WTP threshold. Finally, we exclude grade 1 or 2 adverse events from our analysis due to the assumption that these events have negligible effects on both clinical outcomes and costs. Moreover, our sensitivity analyses confirm the robustness of our findings, as our results remain consistent even when accounting for variations within a range of 25% for grade 3 or higher adverse events. Despite the aforementioned limitations, the study findings remain robust due to the sensitivity analyses conducted. The consistent findings across different sensitivity analyses, where the ICER values remained below the WTP threshold, provide confidence in the cost-effectiveness of the intervention. This demonstrates that even in scenarios where the cost assumptions are modified, the overall conclusion regarding the cost-effectiveness of the intervention remains unchanged.

## 5 Conclusion

Our findings suggest that toripalimab plus chemotherapy in the management of mTNBC is cost-effective compared to placebo plus chemotherapy with an ICER below the WTP threshold in China. These results provide valuable insights for policymakers and healthcare providers in decision-making regarding the optimal treatment strategy for mTNBC patients.

## Data availability statement

The original contributions presented in the study are included in the article/Supplementary material, further inquiries can be directed to the corresponding author.

## Author contributions

ZZ: Validation, Writing – original draft, Writing – review & editing. LH: Data curation, Investigation, Software, Writing – original draft. HC: Project administration, Supervision, Writing – review & editing.
