# Supplementary material for: Toripalimab plus chemotherapy in the treatment of metastatic triple-negative breast cancer: a cost-effectiveness analysis
Source: Front Public Health. 2024 Jul 29;12:1421826. doi: 10.3389/fpubh.2024.1421826 (PMC11317436; doi:10.3389/fpubh.2024.1421826)
Supplement: Supplementary file 1 [file Data_Sheet_1.docx]

**Supplementary Table 1. Comparison of survival models distribution**

|  | AIC | | BIC | |
| --- | --- | --- | --- | --- |
|  | Toripalimab group | Placebo group | Toripalimab group | Placebo group |
| PFS |  |  |  |  |
| Weibull | 754.13 | 409.59 | 760.73 | 414.80 |
| **Log-logistic** | **733.36** | **393.13** | **739.96** | **398.34** |
| Log-normal | 738.73 | 393.03 | 745.33 | 398.24 |
| Gompertz | 754.97 | 414.78 | 761.56 | 419.99 |
| Exponential | 754.20 | 412.86 | 757.50 | 415.46 |
| Gamma | 751.47 | 405.10 | 758.06 | 410.31 |
| OS |  |  |  |  |
| Weibull | 587.71 | 406.97 | 594.31 | 412.18 |
| **Log-logistic** | **584.00** | **401.59** | **590.59** | **406.80** |
| Log-normal | 586.16 | 402.17 | 592.76 | 407.38 |
| Gompertz | 593.16 | 413.15 | 599.75 | 418.36 |
| Exponential | 596.71 | 413.22 | 600.00 | 415.83 |
| Gamma | 586.52 | 404.90 | 593.12 | 410.11 |

AIC: Akaike information criterion; BIC: Bayesian Information Criterion; OS: Overall survival; PFS: Progression-free survival;

**
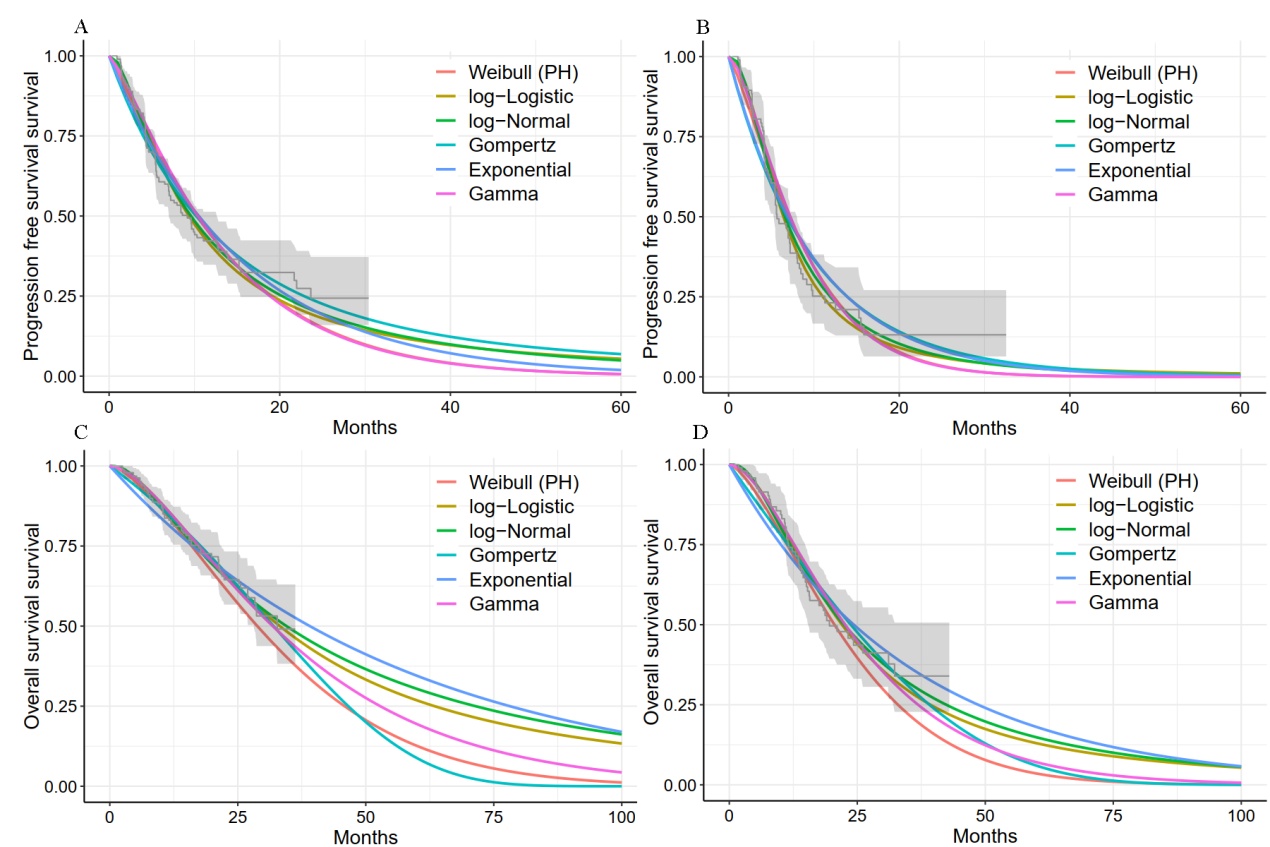
**

**Supplementary Figure 1.** A: Modes simulation visual progression-free survival curve of toripalimab group ; B:Modes simulation visual progression-free survival curve of placebo group; C:Modes simulation visual overall survival curve of toripalimab group; D: Modes simulation visual overall survival curve of placebo group
